# Nanotechnology-enabled immunomodulation in esophageal cancer: targeting the tumor microenvironment to overcome therapeutic barriers

**DOI:** 10.3389/fimmu.2026.1905617

**Published:** 2026-08-14

**Authors:** Qiaoyi Shen, Yibo Gao

**Affiliations:** 1 Department of Thoracic Surgery, National Cancer Center/ National Clinical Research Center for Cancer/ Cancer Hospital, Chinese Academy of Medical Sciences and Peking Union Medical College, Beijing, China; 2 Department of Thoracic Surgery, National Cancer Center/ National Clinical Research Center for Cancer/ Cancer Hospital & Shenzhen Hospital, Chinese Academy of Medical Sciences and Peking Union Medical College, Shenzhen, China; 3 The Affiliated Cancer Hospital, School of Medicine, Southern University of Science and Technology, Shenzhen, China

**Keywords:** cancer immunotherapy, esophageal cancer, immunomodulation, nanomedicine, tumor microenvironment (TME)

## Abstract

Esophageal cancer is a highly aggressive malignancy with a notoriously poor prognosis. While immunotherapy, particularly immune checkpoint blockade (ICB), has revolutionized oncology, its clinical efficacy in esophageal cancer is severely limited by primary and acquired resistance. This resistance is fundamentally driven by a deeply immunosuppressive tumor microenvironment (TME), characterized by severe hypoxia, acidosis, a dense extracellular matrix (ECM), and an abundance of immunosuppressive populations such as M2-like tumor-associated macrophages (TAMs) and myeloid-derived suppressor cells (MDSCs). Consequently, the esophageal TME manifests as an immunologically “cold” phenotype that actively excludes and exhausts effector T cells. Nanotechnology has emerged as a transformative paradigm to dismantle these physiological and immunological barriers. This review provides a comprehensive analysis of nanotechnology-enabled immunomodulation strategies designed to remodel the esophageal TME. We systematically explore state-of-the-art targeted nanoplatforms and highlight their specific mechanisms in TME reprogramming, including alleviating hypoxia and acidosis, repolarizing TAMs, restoring dendritic cell (DC) function, and inducing immunogenic cell death (ICD) and ferroptosis. Furthermore, we evaluate synergistic strategies that combine rationally designed nanomedicines with diverse immunotherapeutic modalities to overcome resistance. By facilitating the crucial transition from “cold” to “hot” tumors, nanotechnology offers promising potential to amplify antitumor immunity and may contribute to improving clinical outcomes in esophageal cancer.

## 1 Introduction

Esophageal cancer remains a lethal malignancy with a dismal prognosis, particularly for advanced esophageal squamous cell carcinoma (ESCC) (1). In recent years, immunotherapy—specifically immune checkpoint inhibitors (ICIs) targeting the Programmed cell death protein 1/Programmed death-ligand 1 (PD-1/PD-L1) axis—has emerged as a transformative clinical paradigm (2). Currently, the combination of PD-1 inhibitors (such as pembrolizumab and nivolumab) with chemotherapy has been established as the standard first-line treatment for advanced esophageal cancer (3). Patient selection for these immunotherapies heavily relies on predictive biomarkers, primarily the PD-L1 Combined Positive Score (CPS) (4). Additionally, high Microsatellite Instability (MSI-H) or deficient Mismatch Repair (dMMR), as well as high Tumor Mutational Burden (TMB), serve as critical biomarkers for identifying patients most likely to achieve favorable and durable responses to immune checkpoint blockade (ICB) (5).

Despite these clinical breakthroughs, the overall efficacy of ICIs in esophageal cancer remains constrained by primary and acquired resistance (6). Primary resistance frequently occurs in patients lacking sufficient tumor immunogenicity or pre-existing T-cell infiltration, or due to the upfront expression of alternative immune checkpoints (e.g., TIM-3, LAG-3) (7). Conversely, patients who initially respond may develop acquired resistance driven by tumor immunoediting, loss of antigen presentation machinery (such as HLA downregulation or B2M mutations), and the progressive accumulation of immunosuppressive factors (8). Fundamentally, both forms of resistance are deeply orchestrated by the dynamic and immunosuppressive nature of the tumor microenvironment (TME) (9).

The esophageal TME acts as a formidable barrier, characterized by hypoxia, acidosis, a dense extracellular matrix (ECM), and a network of immunosuppressive cells including tumor-associated macrophages (TAMs), myeloid-derived suppressor cells (MDSCs), and regulatory T cells (Tregs) (10, 11). Together with metabolic reprogramming that deprives immune effector cells of vital nutrients, these factors collectively create an immune-excluded “cold” tumor phenotype that resists conventional therapies (12–14).

Nanotechnology has emerged as a powerful tool to address these challenges, offering novel strategies for targeted drug delivery, controlled release, and immunomodulation within the TME (15–18). Engineered nanoparticles can be designed to respond to specific TME cues, such as low pH, hypoxia, or elevated reactive oxygen species (ROS) levels, enabling spatiotemporally controlled therapeutic interventions (19–21). Moreover, nanomedicines can co-deliver multiple therapeutic agents, including chemotherapeutic drugs, immunomodulators, and nucleic acids, to simultaneously target different components of the immunosuppressive network (22, 23).

In esophageal cancer, engineered nanomedicines hold particular promise to remodel the TME: they can enhance antigen presentation, promote dendritic cell (DC) maturation, facilitate T cell infiltration, and reprogram immunosuppressive cells while minimizing systemic toxicity (15, 19, 21, 24, 25). This review provides a comprehensive analysis of nanotechnology-enabled immunomodulation strategies in esophageal cancer. We examine the immunosuppressive TME landscape, explore various targeted nanomaterial platforms and TME reprogramming approaches, and evaluate synergistic strategies combining nanomedicine with immunotherapies to overcome resistance and improve clinical outcomes.

To ensure a comprehensive and systematic evaluation of the field, a rigorous literature search strategy was employed for this review. We searched electronic databases including PubMed, Web of Science, and Scopus for peer-reviewed articles published from inception to May 2026. The search was conducted using combinations of the following keywords: (‘esophageal cancer’ OR ‘esophageal squamous cell carcinoma’ OR ‘ESCC’ OR ‘esophageal adenocarcinoma’) AND (‘tumor microenvironment’ OR ‘TME’ OR ‘immunotherapy’ OR ‘immune checkpoint blockade’) AND (‘nanomedicine’ OR ‘nanoparticles’ OR ‘nanomaterials’ OR ‘drug delivery’). Study selection was limited to peer-reviewed original research articles and authoritative reviews published in English. Studies primarily focusing on nanotechnology-based modulation of the esophageal tumor microenvironment and those exploring synergistic immuno-nanotherapeutic strategies were prioritized for inclusion, while conference abstracts, unpublished data, and non-English publications were excluded.

## 2 The esophageal cancer TME: a landscape of immunosuppressive barriers

**Figure 1:**
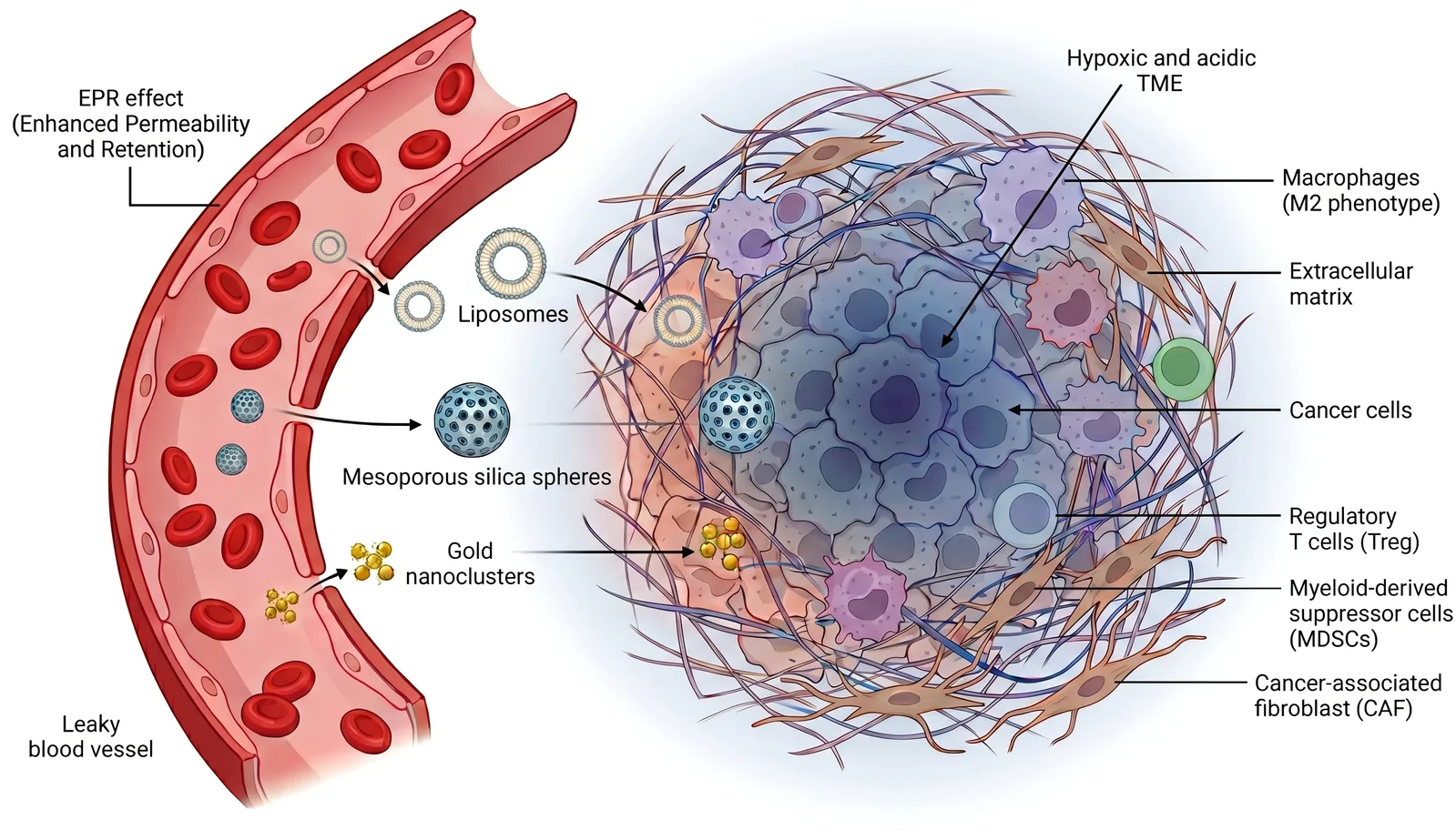
The immunosuppressive landscape of the esophageal cancer TME and the EPR effect. Leaky tumor vasculature allows for the extravasation and accumulation of various nanocarriers (e.g., liposomes, mesoporous silica spheres, and gold nanoclusters) via the EPR effect. It is critical to note that this EPR-mediated accumulation is highly heterogeneous and non-uniform in human tumors. Rather than a deterministic process, extravasation is frequently hindered by the dense ECM constructed by CAFs and the resulting high interstitial fluid pressure. Within the tumor parenchyma, a deeply immunosuppressive niche is established by a dense ECM constructed by cancer-associated fibroblasts (CAFs), alongside core regions characterized by severe hypoxia and acidosis. This physical and metabolic barrier is further reinforced by a network of immunosuppressive cells, including M2-like macrophages, Tregs, and MDSCs, which collectively protect cancer cells from immune clearance.

The TME in esophageal cancer is a complex ecosystem comprising tumor cells, immune cells, stromal cells, signaling molecules, and ECM components that collectively contribute to immunosuppression and therapeutic resistance (10, 26). Understanding the cellular and molecular composition of this microenvironment is essential for developing effective nanotechnology-based immunomodulatory strategies (**Figure 1**). Before delving into the specific immunosuppressive barriers, it is critical to distinguish between the two primary histological subtypes of esophageal cancer: esophageal squamous cell carcinoma (ESCC) and esophageal adenocarcinoma (EAC). These two subtypes are fundamentally distinct diseases. Epidemiologically, ESCC is predominant in Asian and African populations and is strongly associated with smoking and alcohol consumption, whereas EAC is more prevalent in Western countries and is closely linked to gastroesophageal reflux disease (GERD), Barrett’s esophagus, and obesity. Molecularly and immunologically, they also exhibit distinct landscapes. ESCC is typically characterized by frequent TP53 mutations, genomic instability, and a TME densely infiltrated with immunosuppressive cells such as M2-like TAMs and exhausted T cells, often showing a stronger rationale for PD-1/PD-L1 blockade. Conversely, EAC often shares molecular similarities with the chromosomal instability subtype of gastric cancer and presents a TME heavily influenced by chronic inflammation and a distinct fibrotic stroma. It should be noted that the majority of current preclinical nanomedicine studies and *in vivo* models discussed in this review primarily focus on ESCC. However, the overarching principles of nanotechnology-enabled TME reprogramming—such as alleviating hypoxia, neutralizing acidosis, and remodeling the extracellular matrix—are highly applicable to both subtypes, provided that the nanocarriers are rationally designed to target their specific biological contexts.

Hypoxia represents a hallmark feature of the esophageal TME, resulting from the imbalance between rapid tumor cell proliferation and inadequate vascular supply (27, 28). Hypoxia-inducible factor-1α (HIF-1α) activation drives multiple immunosuppressive pathways, including the recruitment of MDSCs and TAMs, promotion of M2 macrophage polarization, and upregulation of immune checkpoint molecules (27, 28). The hypoxic environment also impairs cytotoxic T cell function and promotes T cell exhaustion, creating a self-reinforcing cycle of immunosuppression (27, 29). Furthermore, hypoxia contributes to the development of resistance to both chemotherapy and immunotherapy by activating survival pathways and promoting genetic instability (14, 28).

The acidic TME, resulting from enhanced glycolysis and lactate production by tumor cells (the Warburg effect), further suppresses antitumor immunity (30, 31). Low extracellular pH impairs the function of infiltrating T cells and natural killer (NK) cells while promoting the activity of immunosuppressive cells such as TAMs and MDSCs (30, 31). The acidic environment also limits the efficacy of therapeutic antibodies and checkpoint inhibitors by affecting their binding affinity and stability (21, 29).

TAMs constitute the most abundant immune cell population within the esophageal TME and play a pivotal role in driving immunosuppression (11, 32). Historically, TAMs have been broadly classified into a binary framework: the antitumor M1-like phenotype and the protumor M2-like phenotype (11, 33). However, it is now widely recognized that this binary classification is an oversimplification. *In vivo*, TAMs exhibit remarkable plasticity and exist along a highly dynamic phenotypic continuum, adapting constantly to diverse cues within the TME (34). Furthermore, TAM populations demonstrate significant spatial heterogeneity; for instance, macrophages residing in the hypoxic core of the tumor often display distinctly different transcriptomic and functional profiles compared to those at the well-oxygenated invasive margin (34). Nevertheless, for the conceptual purpose of defining therapeutic targets, it remains clinically relevant that within this continuum, TAMs in ESCC predominantly skew toward an immunosuppressive, M2-like functional state, secreting cytokines such as IL-10 and TGF-β, promoting angiogenesis, and facilitating tumor invasion and metastasis (11, 32, 33). The accumulation of M2-like TAMs correlates with poor prognosis and resistance to immunotherapy in ESCC patients (32, 35). TAMs also contribute to immune evasion by expressing PD-L1 and other immune checkpoint molecules that directly inhibit T cell function (11, 36).

MDSCs represent another critical immunosuppressive population in the esophageal TME (10, 37). MDSCs expand in response to tumor-derived factors and accumulate in both the tumor tissue and peripheral blood of cancer patients (37). These cells suppress T cell activation through multiple mechanisms, including the depletion of arginine and cysteine, production of ROS and reactive nitrogen species, and secretion of immunosuppressive cytokines (37, 38). MDSCs also promote the expansion of Tregs and inhibit DC maturation, further amplifying the immunosuppressive network (10, 37).

Cancer-associated fibroblasts (CAFs) constitute a major stromal component of the esophageal TME and contribute significantly to immune exclusion and therapeutic resistance (39–41). CAFs secrete ECM components, including collagen and hyaluronan, that form a physical barrier impeding immune cell infiltration and drug penetration (39, 41). Additionally, CAFs produce immunosuppressive factors such as CXCL12, TGF-β, and IL-6 that recruit and activate immunosuppressive cells while excluding effector T cells from the tumor parenchyma (39, 41, 42). The CXCL12-CXCR4 axis, in particular, plays a crucial role in maintaining the immunosuppressive niche by promoting TAM recruitment and inhibiting CD8+ T cell migration (42, 43).

The ECM itself presents a formidable barrier to immunotherapy in esophageal cancer (29, 44). The dense, fibrotic ECM not only physically excludes immune cells but also creates a biomechanical microenvironment that modulates immune cell function (29). Matrix stiffness has been shown to influence macrophage polarization toward the M2 phenotype and impair T cell motility and effector function (29, 41). The ECM also sequesters growth factors and cytokines that sustain the immunosuppressive milieu (29, 44).

DCs, the most potent antigen-presenting cells (APCs), are often dysfunctional in the esophageal TME (24, 45). Tumor-derived factors impair DC maturation, antigen uptake, and migration to lymph nodes, resulting in inadequate T cell priming (24, 45). The immunosuppressive TME also promotes the accumulation of tolerogenic DCs that induce T cell anergy and promote Treg expansion (45). These defects in DC function represent a critical bottleneck in the cancer-immunity cycle that limits the efficacy of immunotherapy (21, 24).

The immunosuppressive landscape of the esophageal TME is further reinforced by metabolic competition between tumor cells and immune cells (12, 13). Tumor cells consume large quantities of glucose and glutamine, depriving T cells of the metabolic resources required for their activation and proliferation (12, 13). The accumulation of lactate, kynurenine, and other metabolic byproducts within the TME directly suppresses T cell function and promotes the differentiation of immunosuppressive cell populations (12, 13, 46). This metabolic reprogramming creates a hostile environment that favors tumor progression while inhibiting effective antitumor immunity.

## 3 Nanomaterial platforms for targeted delivery and modulation in esophageal cancer

Nanotechnology offers a diverse array of material platforms that can be engineered to overcome the biological barriers presented by the esophageal TME (47–49). These nanocarriers provide unique advantages, including enhanced drug solubility, prolonged circulation time, targeted delivery, and controlled release of therapeutic payloads (16, 17, 50). Historically, the accumulation of these nanocarriers in the TME has heavily relied on the enhanced permeability and retention (EPR) effect. However, a critical translational challenge is that while the EPR effect works robustly in preclinical murine models, it is highly heterogeneous and substantially less predictable in human patients (51, 52). In human esophageal cancer, the dense desmoplastic stroma and the resulting high interstitial fluid pressure (IFP) act as formidable physical barriers, often preventing the uniform extravasation and deep tissue penetration of nanocarriers. Consequently, modern nanomedicine designs increasingly incorporate active targeting ligands, TME-responsive elements, or stroma-modulating agents to actively overcome the limitations of passive, EPR-driven delivery.

Lipid-based nanoparticles, including liposomes and lipid nanoparticles (LNPs), represent one of the most clinically advanced nanocarrier platforms for cancer therapy (48, 53). These systems can encapsulate both hydrophilic and hydrophobic drugs, protect nucleic acid cargoes from enzymatic degradation, and facilitate cellular uptake through fusion with cell membranes (53, 54). LNPs have been successfully employed for the delivery of small interfering RNA (siRNA) and messenger RNA (mRNA) in cancer immunotherapy (53–55). In the context of ESCC, lipid-based nanocarriers can be engineered to co-deliver chemotherapeutic agents and immunomodulatory molecules, enabling synergistic chemo-immunotherapy (56, 57).

Polymeric nanoparticles, composed of biodegradable polymers such as PLGA, PEG-PLGA, or chitosan, offer excellent biocompatibility and tunable drug release profiles (47, 48). These platforms can be surface-functionalized with targeting ligands, including antibodies, peptides, or aptamers, to achieve active targeting of specific cell populations within the TME (47, 50, 58). pH-responsive polymeric nanoparticles have been designed to release their payloads selectively in the acidic TME, minimizing systemic toxicity while maximizing therapeutic efficacy at the tumor site (19, 21, 59). Polymeric micelles, formed through self-assembly of amphiphilic block copolymers, can encapsulate hydrophobic drugs and improve their bioavailability (48, 56).

Mesoporous silica nanoparticles (MSNs) have attracted considerable attention as versatile drug delivery platforms due to their high surface area, tunable pore size, and ease of surface modification (60, 61). MSNs can be loaded with multiple therapeutic agents, including chemotherapeutic drugs, photosensitizers, and immunomodulators, enabling multimodal therapy (60, 61). The mesoporous structure protects cargo molecules from premature degradation and allows for sustained release (60). Manganese-doped MSNs have been developed to regulate the TME through glutathione depletion, ROS generation, and oxygenation, thereby enhancing the efficacy of chemo-immunotherapy (61).

Metal-based nanoparticles, including gold nanoparticles, iron oxide nanoparticles, and manganese dioxide (MnO_2_) nanoparticles, offer unique physicochemical properties that can be exploited for both therapeutic and diagnostic applications (20, 62, 63). MnO_2_ nanoparticles have been extensively studied for TME modulation due to their ability to generate oxygen from endogenous hydrogen peroxide (H_2_O_2_), thereby alleviating tumor hypoxia (62, 64). MnO_2_-based nanosystems can also deplete glutathione, enhance ROS production, and activate the cGAS-STING pathway, promoting antitumor immune responses (62, 65). Bismuth-based nanoparticles have been developed for radiosensitization and miRNA delivery in ESCC, demonstrating the potential of metal-based platforms for combined therapy (66).

Metal-phenolic networks (MPNs), formed through coordination between phenolic ligands and metal ions, have emerged as a versatile platform for TME-responsive drug delivery (67). These systems exhibit pH-dependent disassembly, enabling controlled release of therapeutic agents in the acidic TME (67). MPNs can be engineered to incorporate multiple functional components, including photosensitizers, chemotherapeutic drugs, and immunomodulators, for synergistic cancer therapy (67, 68).

As an early-stage proof-of-concept, biomimetic nanoparticles, which leverage natural cell membranes or extracellular vesicles for surface coating, represent an innovative approach to overcome biological delivery barriers (15, 69). Coating nanoparticles with cancer cell membranes enables homologous targeting, while macrophage membrane coating facilitates immune evasion and enhanced tumor accumulation (15, 69). Hybrid cellular nanovesicles, generated by fusing membranes from different cell types, can combine multiple functionalities within a single platform (69). For example, hybrid nanovesicles derived from M1 macrophages and PD-1-overexpressing tumor cells can simultaneously repolarize TAMs and block PD-1/PD-L1 interactions (69).

Stimuli-responsive nanocarriers, designed to release their payloads in response to specific TME cues, offer precise spatiotemporal control over drug delivery (19, 21, 70). pH-responsive systems exploit the acidic TME to trigger drug release, while redox-responsive carriers utilize the elevated glutathione levels in tumor cells for intracellular drug release (19, 21, 71). Hypoxia-responsive nanoplatforms can be activated under low oxygen conditions, enabling targeted therapy in the hypoxic tumor core (27, 57). Light-responsive nanomedicines, activated by external light sources, offer the advantage of remote control over drug release and therapeutic activity (25, 56, 72).

Exosomes and other extracellular vesicles represent naturally derived nanocarriers that have gained attention for cancer immunotherapy (15, 53). These biological vesicles can be loaded with therapeutic cargoes and engineered to display targeting ligands on their surface (15, 53). M1 macrophage-derived extracellular vesicles have been shown to promote M2-to-M1 macrophage repolarization and enhance antitumor immunity (73). Exosomes can also be combined with synthetic nanoparticles to create hybrid delivery systems that leverage the advantages of both natural and synthetic platforms (15).

The selection of appropriate nanomaterial platforms for esophageal cancer immunotherapy depends on multiple factors, including the specific therapeutic cargo, the target cell population, and the desired release kinetics (48, 50). The physicochemical properties of nanoparticles, including size, shape, surface charge, and ligand density, critically influence their biodistribution, cellular uptake, and therapeutic efficacy (50, 74). Optimizing these parameters is essential for achieving effective TME modulation and immunotherapeutic outcomes in esophageal cancer. A comprehensive summary of representative nanotechnology-enabled immunomodulation strategies, including their targeted mechanisms, specific cancer models, and translational limitations, is provided in **Table 1**.

**Table 1:** Representative nanotechnology-enabled immunomodulation strategies and their translational landscape.

Nanoplatform	Payload(s)	Immune/stromal target	Cancer model	Principal findings	Evidence level	Translational limitations
Mesoporous Silica (Ce6-SiO2@MnO2) on Stents	Ce6 (photosensitizer), MnO2	Hypoxia relief, TAMs (M1 repolarization)	ESCC	Reversed hypoxic TME; reprogrammed TAMs via Mn2+ signaling; enhanced antitumor immunity; improved stent patency.	Preclinical (*in vivo*)	Potential heavy metal accumulation and toxicity; complex manufacturing process for stent coating.
Bismuth Sulfide Nanoflowers	miR339	Cancer Stemness, Radiosensitization	ESCC	Facilitated targeted miRNA delivery; overcame stemness and radioresistance via USP8 inhibition.	Preclinical (*in vivo*)	Long-term biocompatibility and clearance mechanisms of bismuth nanomaterials remain to be fully elucidated.
Lipid Nanocapsules (LNCs@si-CXCL12)	siRNA targeting CXCL12	CAFs, MDSCs	ESCC (Spontaneous model)	Silenced CXCL12 expression in CAFs; reduced MDSC recruitment; restored CD8+ T cell function.	Preclinical (*in vivo*)	Challenges in systemic siRNA stability; maintaining targeting specificity to CAFs without off-target effects.
Albumin-bound Nanoparticles (nab-paclitaxel)	Paclitaxel	Tumor cells (ICD induction), Exhausted T cells	ESCC	Combined with PD-1 blockade for synergistic chemo-immunotherapy; achieved a major pathological response rate of 62% in a neoadjuvant setting.	Clinical (Phase II Trial)	Potential systemic toxicities; lack of definitive predictive biomarkers to accurately identify responders.
Light-switchable Micelles	Paclitaxel, Verteporfin	ICD induction, Angiogenesis (VEGF)	ESCC	Induced robust ICD; inhibited VEGF-mediated angiogenesis; enabled dual chemo-photodynamic immunotherapy.	Preclinical (*in vivo*)	Limited tissue penetration depth of external light sources for deep-seated or metastatic esophageal tumors.
Polymeric Nanoparticles	CXCR4 antagonist	TAMs (M1 repolarization)	Hepatocellular Carcinoma (HCC)	Blocked CXCL12/CXCR4 axis; inhibited Akt/mTOR; repolarized M2-like TAMs to M1 phenotype.	Preclinical (*in vivo*)	Requires direct validation within the uniquely dense and highly fibrotic stroma of esophageal cancer.
CaCO3-complexed pH-responsive Nanoparticles	Mitoxantrone, Celastrol	Acidic TME neutralization, ICD	Colon Cancer	Consumed H+ to neutralize TME acidosis; promoted robust immunogenic cell death.	Preclinical (*in vivo*)	High variability in TME acidity among patients; lacks direct validation in esophageal tumor models.
Biomimetic Hybrid Nanovesicles	M1 macrophage membrane, PD-1 overexpressing membrane	TAMs (M1 repolarization), PD-1/PD-L1 axis	Cancer Model	Simultaneously repolarized TAMs and blocked immune checkpoints for a synergistic antitumor effect.	Preclinical (*in vivo*)	Scalability issues in extracting and fusing cell membranes; potential long-term immunogenicity of hybrid membranes.

## 4 Nanotechnology-enabled reprogramming of TME components for immunomodulation

**Figure 2:**
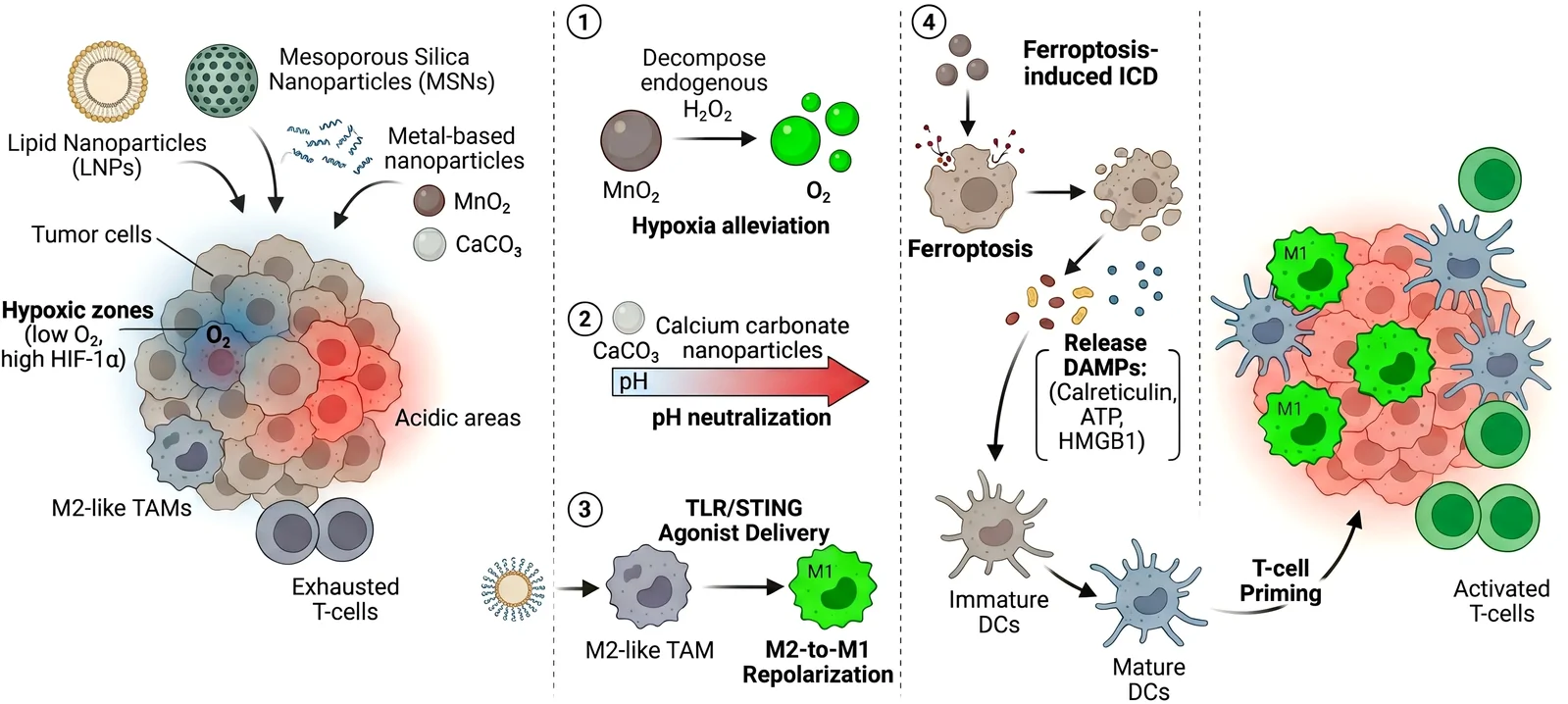
Mechanistic strategies for nanotechnology-enabled reprogramming of the immunosuppressive TME. Engineered nanocarriers, including LNPs, MSNs, and metal-based nanoparticles, can be rationally designed to overcome specific TME barriers. (1) MnO₂ nanoparticles alleviate tumor hypoxia by catalyzing the decomposition of endogenous H₂O₂ into oxygen (O₂). (2) Calcium carbonate (CaCO₃) nanoparticles act as proton sponges to neutralize the acidic TME. (3) Targeted delivery of TLR/STING agonists repolarizes protumor M2-like TAMs into the antitumor M1-like phenotype. (4) Nanoparticle-induced ferroptosis can triggers immunogenic cell death (ICD), leading to the robust release of damage-associated molecular patterns (DAMPs), such as calreticulin, ATP, and HMGB1. Importantly, rather than an inevitable linear sequence, this immunostimulatory cascade is highly context-dependent. Under optimal spatiotemporal conditions, these signals promote the maturation of DCs and facilitate robust T-cell priming; however, uncontrolled or prolonged ferroptosis may alternatively generate oxidized lipids that promote immunosuppression.

Nanotechnology provides powerful tools for reprogramming the immunosuppressive TME in esophageal cancer through targeted modulation of specific cellular and molecular components (15, 16, 75). These strategies aim to convert the immunologically “cold” TME into a “hot” TME that supports effective antitumor immunity (14, 76) (**Figure 2**).

Repolarization of TAMs from the protumor M2-like phenotype to the antitumor M1-like phenotype represents a major focus of nanotechnology-enabled immunomodulation (11, 33, 35, 36). Various nanoparticle formulations have been developed to deliver immunostimulatory agents that promote M1 polarization, including Toll-like receptor (TLR) agonists, interferon-γ, and NF-κB activators (11, 36, 77), though these strategies predominantly remain at the *in vivo* animal testing stage. Manganese ions released from MnO_2_ nanoparticles can reprogram TAMs toward the M1 phenotype by activating the STING pathway and promoting pro-inflammatory cytokine production (62, 64). In a study involving ESCC, Ce6-SiO_2_@MnO_2_ nanoparticles loaded onto esophageal stents effectively reversed the hypoxic TME and reprogrammed TAMs through Mn2+-mediated signaling, resulting in enhanced antitumor immunity and improved stent patency (64).

Nanoparticles delivering small molecule inhibitors or nucleic acids can directly suppress M2 polarization pathways (33, 36). CXCR4 antagonist-loaded nanoparticles have been shown to block the CXCL12/CXCR4 axis, inhibiting Akt and mTOR activation in TAMs and promoting their repolarization toward the M1 phenotype (43). siRNA-loaded nanoparticles targeting immunosuppressive genes in TAMs offer another approach for phenotype reprogramming (33, 78). The combination of TAM repolarization with other immunotherapeutic strategies, such as immune checkpoint blockade, can produce synergistic antitumor effects (36, 69).

Depletion of immunosuppressive TAMs represents an alternative strategy for TME reprogramming (11, 35, 36). Nanoparticles can deliver cytotoxic agents selectively to TAMs by exploiting their phagocytic activity or by targeting surface receptors such as CD206 or CSF1R (35, 36). Bisphosphonate-loaded nanoparticles have been used to deplete TAMs in preclinical models, resulting in reduced tumor growth and enhanced T cell infiltration (35). However, careful consideration must be given to the potential loss of antitumor macrophages that may also be affected by depletion strategies (11, 36).

Targeting MDSCs is another critical approach for reversing immunosuppression in the esophageal TME (37, 38). Nanoparticles can be designed to inhibit MDSC recruitment, promote their differentiation into mature myeloid cells, or selectively eliminate MDSC populations (37, 38). LNPs delivering siRNA against immunosuppressive factors such as CXCL12 can reduce MDSC accumulation and restore CD8+ T cell function (42). In a spontaneous ESCC mouse model, LNCs@si-CXCL12 nanoparticles effectively silenced CXCL12 expression in CAFs, reducing MDSC recruitment and enhancing antitumor immunity (42).

Modulation of CAFs represents a promising but challenging strategy for TME reprogramming in esophageal cancer (39–41). CAFs contribute to immune exclusion through ECM deposition and secretion of immunosuppressive factors (39, 41). Nanoparticles targeting fibroblast activation protein (FAP), a marker expressed on CAFs, can deliver therapeutic agents specifically to these cells (39, 40). CAF-targeted nanoparticles loaded with chemotherapeutic agents or signaling inhibitors can reduce CAF density, normalize ECM architecture, and improve immune cell infiltration (39, 41). The combination of CAF modulation with ICIs has shown synergistic effects in preclinical models (39, 41).

Restoring DC function is essential for effective T cell priming and antitumor immunity (24, 45). Nanoparticles can enhance DC maturation and antigen presentation through several mechanisms (15, 45). Delivery of TLR agonists, STING agonists, or other adjuvants via nanoparticles can activate DCs and promote their migration to lymph nodes (24, 45, 79). Nanovaccines, which co-deliver tumor antigens and adjuvants within a single nanoparticle platform, can efficiently target DCs and induce robust T cell responses (53, 60, 80). The size, shape, and surface properties of nanovaccines can be optimized to enhance DC uptake and cross-presentation of antigens (60, 80).

Alleviating tumor hypoxia is a key strategy for reversing multiple immunosuppressive mechanisms in the TME (27, 28). MnO_2_-based nanoparticles can catalyze the decomposition of endogenous H_2_O_2_ to generate oxygen, thereby relieving hypoxia and reducing HIF-1α expression (62, 64). Oxygen-generating nanoparticles have been shown to enhance the efficacy of photodynamic therapy (PDT) and radiotherapy in experimental tumor models by improving ROS production and reducing hypoxia-mediated resistance (27, 62, 72). Hypoxia relief also promotes T cell infiltration and function while reducing the accumulation of immunosuppressive cells (27, 28).

Normalization of the acidic TME can enhance antitumor immunity by improving immune cell function and facilitating drug delivery (30, 31). Calcium carbonate (CaCO_3_) nanoparticles can neutralize acidic pH through proton consumption, thereby alleviating TME acidosis (31, 81, 82). CaCO_3_-complexed pH-responsive nanoparticles encapsulating mitoxantrone and celastrol have been shown to reprogram the TME by consuming H+ and promoting immunogenic cell death (ICD) (82). A hierarchically structured fiber device incorporating proton pump inhibitors and M1 macrophage membrane-coated nanoparticles effectively restored TME pH from 6.8 to 7.2 and enhanced antitumor immune responses (31).

Metabolic reprogramming within the TME offers additional targets for nanotechnology-enabled immunomodulation (12, 13, 46, 83). Nanoparticles can deliver inhibitors of glycolysis, glutamine metabolism, or tryptophan catabolism to disrupt the metabolic advantage of tumor cells and restore immune cell function (12, 13, 46). Indoleamine 2,3-dioxygenase (IDO) inhibitors, which block kynurenine production, can be delivered via nanoparticles to relieve T cell suppression (84). Dual-regulated biomimetic nanocomposites combining photodynamic therapy with IDO inhibition have shown enhanced antitumor immunity by breaking the “immune-metabolism” loop in melanoma and breast cancer models (84). Translating these metabolic-modulating nanomedicines to esophageal cancer, a tumor heavily characterized by lactate accumulation and glucose deprivation, represents a highly promising future direction.

Induction of immunogenic cell death represents a powerful strategy for converting the immunosuppressive TME into an immunostimulatory environment (72, 85). ICD is characterized by the release of damage-associated molecular patterns (DAMPs), including calreticulin exposure, ATP secretion, and HMGB1 release, which activate DCs and promote T cell priming (72, 85, 86). Various nanotechnology platforms have been developed to induce ICD through chemotherapy, photodynamic therapy, photothermal therapy, or ferroptosis (72, 85–87). The combination of ICD-inducing nanoparticles with ICIs can produce synergistic antitumor effects by simultaneously promoting T cell priming and blocking immune evasion (85, 86).

Ferroptosis, an iron-dependent form of regulated cell death, has emerged as a promising strategy for enhancing cancer immunotherapy (86, 88–90). Ferroptosis-inducing nanoparticles can trigger ICD and promote antitumor immune responses through the release of DAMPs and the activation of innate immune pathways (65, 86, 88). However, it is crucial to recognize that ferroptosis is not invariably immunogenic. Recent evidence indicates that its impact on the TME is highly context-dependent and acts as a double-edged sword (91). While early-stage ferroptosis can stimulate antitumor immunity, the accumulation of oxidized lipids and the subsequent release of specific signaling molecules, such as prostaglandin E2 (PGE2), from ferroptotic cancer cells can paradoxically promote immunosuppression (91, 92). These lipid mediators can recruit suppressive TAMs, impair DC cross-presentation, and directly compromise CD8+ T cell effector function. Therefore, the rational design of ferroptosis-inducing nanomedicines must meticulously consider the timing, dosage, and specific TME context to tip the balance toward immunostimulation rather than immunosuppression. Fe3+-doped dendritic mesoporous organosilica nanoparticles have been designed as ferroptosis inducers that generate hydroxyl radicals through Fenton reactions and deplete glutathione, triggering lethal lipid peroxidation and ICD (93). The combination of ferroptosis-inducing nanoparticles with ICIs promotes long-term antitumor immune responses and prevents metastasis (65, 93).

## 5 Synergistic strategies: combining nanomedicine with immunotherapies to overcome resistance

The combination of nanomedicine with established immunotherapeutic modalities offers synergistic potential for overcoming resistance mechanisms in esophageal cancer (17, 22, 75, 94). However, it must be emphasized that with the exception of certain clinically approved formulations (e.g., nab-paclitaxel), the vast majority of these sophisticated, multi-component synergistic nanoplatforms are currently confined to early preclinical investigation. These combination strategies aim to simultaneously address multiple barriers in the cancer-immunity cycle, from antigen release and presentation to T cell activation and effector function (21, 24).

Nanoparticle-mediated delivery of ICIs represents a promising approach to enhance the efficacy of ICB therapy while reducing systemic toxicity (94–96). PD-1/PD-L1 blockade can be potentiated by nanoparticles that modulate the TME to promote T cell infiltration and activation (81, 95). pH-sensitive nanoparticles co-delivering anti-PD-1 antibodies and siRNA against midkine (MDK) have been shown to overcome ICB resistance in hepatocellular carcinoma by reprogramming TAMs and MDSCs, demonstrating the potential of this approach for esophageal cancer (96). Engineered PD-L1 nanoregulators, including nanoparticles that downregulate PD-L1 expression or block PD-1/PD-L1 interactions, offer alternative strategies for enhancing ICB therapy (95).

The combination of photodynamic therapy with immunotherapy has attracted considerable attention for converting “cold” tumors to “hot” tumors (25, 56, 72). PDT induces ICD through ROS-mediated cell death, releasing tumor antigens that activate DCs and promote T cell priming (56, 72). However, the immunosuppressive TME often limits the efficacy of PDT-based immunotherapy (72). Nanoplatforms that combine photosensitizers with immunomodulatory agents can simultaneously induce ICD and reprogram the TME, producing synergistic antitumor effects (56, 72). In ESCC, a nanoengineered light-switchable micelle system co-delivering paclitaxel and verteporfin demonstrated dual chemo-photodynamic immunotherapy by inducing ICD and inhibiting VEGF-mediated angiogenesis (56).

Photothermal therapy (PTT) combined with immunotherapy offers another synergistic approach for ESCC treatment (48, 97). Near-infrared (NIR) dye-based nanoparticles can mediate PTT while also serving as carriers for immunostimulatory agents (97). TME-targeting amphiphilic NIR cyanine nanoparticles loaded with CpG adjuvant have been shown to induce ICD and promote CD8+ T cell infiltration when combined with laser irradiation (97). The addition of immune checkpoint blockade further enhanced systemic antitumor immunity, inhibiting both primary and distant tumor growth (97).

Chemo-immunotherapy, combining cytotoxic chemotherapy with immunotherapy, represents a clinically translatable strategy for ESCC (23, 59, 98). Chemotherapeutic agents can induce ICD, promoting antigen release and DC activation (23, 85). However, chemotherapy can also exacerbate immunosuppression by depleting immune cells and promoting MDSC accumulation (23). Nanoparticle-based co-delivery systems can simultaneously deliver chemotherapeutic agents and immunomodulators to achieve synergistic effects while minimizing systemic toxicity (23, 59). When evaluating clinical progress, it is important to carefully distinguish between first-generation nanocarriers and next-generation, actively TME-remodeling platforms. In a Phase II clinical trial for ESCC, neoadjuvant chemo-immunotherapy combining PD-1 blockade with nanoparticle albumin-bound paclitaxel (nab-paclitaxel) demonstrated promising efficacy, with a major pathological response rate of 62% (98). However, it must be noted that nab-paclitaxel primarily utilizes the albumin nanocarrier to improve drug solubility and abrogate solvent-related toxicities, rather than to specifically and actively remodel the TME. The induction of ICD in this clinical context is predominantly driven by the chemotherapeutic payload (paclitaxel) itself. Nevertheless, the clinical success of this regimen provides a strong baseline and proof-of-concept. It underscores the immense translational feasibility of combining nanocarrier-delivered therapies with immune checkpoint blockade, thereby paving the way for more advanced, rationally engineered nanomedicines that can actively reprogram the TME to enter clinical evaluation.

STING agonists have emerged as powerful immunostimulatory agents that activate innate immunity and promote antitumor T cell responses (79, 99–101). However, systemic administration of STING agonists is limited by poor bioavailability and off-target toxicity (79, 100). Nanoparticle encapsulation of STING agonists can overcome these limitations by enabling targeted delivery and controlled release (79, 100, 101). STING-activating nanoparticles have been shown to reprogram the immunosuppressive TME by promoting DC activation, cytotoxic T cell priming, and macrophage re-education (79, 100). The combination of STING-activating nanomedicines with ICIs produces synergistic antitumor effects in preclinical models (99–101). Although direct validation in esophageal cancer models is currently sparse, these established frameworks strongly suggest that STING-targeted nanodelivery could effectively overcome the ‘cold’ immune phenotype typical of the esophageal TME.

Cancer vaccines, including neoantigen-based vaccines, represent a personalized approach to cancer immunotherapy (53, 55, 80, 102). Nanotechnology can enhance vaccine efficacy by protecting antigens from degradation, promoting DC uptake, and providing adjuvant activity (53, 80, 102). LNPs have been successfully employed for mRNA vaccine delivery, as demonstrated by the clinical development of autogene cevumeran (BNT122) for pancreatic cancer (55). For ESCC, nanovaccines targeting specific tumor antigens or neoantigens could potentially induce durable antitumor immunity (80, 102). *In situ* vaccination strategies using nanomaterials that induce ICD and capture tumor antigens for DC presentation offer a personalized approach (demonstrated primarily in melanoma and colon cancer models) without requiring prior antigen identification (103, 104). Applying these nanotechnology-driven *in situ* vaccination strategies to esophageal cancer remains a largely unexplored but clinically vital frontier.

Chimeric antigen receptor (CAR)-T cell therapy has achieved remarkable success in hematologic malignancies but faces significant challenges in solid tumors, including esophageal cancer (105–108). The immunosuppressive TME limits CAR-T cell infiltration, survival, and effector function (105–107). Nanotechnology offers multiple strategies to enhance CAR-T cell therapy, including nanoparticle-mediated CAR delivery to T cells *in vivo*, nanoparticle-based modulation of the TME to support CAR-T cell function, and nanoparticle-enabled real-time monitoring of CAR-T cell activity (105, 106, 108). CAR-macrophage therapy represents an emerging approach that combines the tumor-homing properties of macrophages with CAR-mediated tumor recognition (105, 108, 109).

Radiotherapy combined with immunotherapy can produce synergistic antitumor effects through the abscopal effect, where local irradiation induces systemic antitumor immunity (110–112). However, the immunosuppressive TME often limits the efficacy of radio-immunotherapy (110, 111). Nanomedicine can enhance radio-immunotherapy by delivering radiosensitizers that amplify DNA damage and ICD, while simultaneously delivering immunomodulators that reprogram the TME (110–112). Ultrasound-activated nanoplatforms that induce multiple modes of cell death, including apoptosis, ferroptosis, and ICD, have been shown to reshape the TME and enhance the efficacy of immune checkpoint blockade (110).

Metabolic modulation combined with immunotherapy represents an emerging strategy for overcoming resistance in esophageal cancer (12, 13, 46, 83). Nanomedicines targeting metabolic pathways, including glycolysis, amino acid metabolism, and lipid metabolism, can reprogram the TME to support antitumor immunity (12, 13, 46). The combination of metabolic modulators with ICIs or cancer vaccines can produce synergistic effects by simultaneously depriving tumor cells of metabolic resources and activating antitumor immune responses (46, 83).

## 6 Conclusion and outlook

Nanotechnology represents a highly promising paradigm for overcoming the immunosuppressive barriers that limit the efficacy of immunotherapy in esophageal cancer. The complex and dynamic TME, characterized by hypoxia, acidosis, dense ECM, and diverse immunosuppressive cell populations, presents multiple targets for nanotechnology-enabled intervention. Engineered nanocarriers can deliver therapeutic payloads with spatiotemporal precision, modulate specific cellular populations, and reprogram the TME to support effective antitumor immunity.

Significant progress has been made in developing nanomaterial platforms for TME modulation, including LNPs, polymeric nanoparticles, MSNs, metal-based nanoparticles, and biomimetic systems. These platforms can be engineered to respond to specific TME cues, enabling controlled drug release at tumor sites. The ability to co-deliver multiple therapeutic agents within a single nanocarrier allows for simultaneous targeting of different immunosuppressive mechanisms, producing synergistic antitumor effects.

Nanotechnology-enabled strategies for TME reprogramming include repolarization of TAMs, depletion of MDSCs, modulation of CAFs, restoration of DC function, alleviation of hypoxia and acidosis, and induction of ICD and ferroptosis. These approaches can convert the immunologically “cold” TME into a “hot” TME that supports T cell infiltration and effector function. Combination strategies that integrate nanomedicine with ICIs, photodynamic therapy, chemotherapy, radiotherapy, or CAR-T cell therapy offer the potential for enhanced therapeutic efficacy and durable antitumor immunity.

Despite these promising advances, several challenges must be addressed before nanotechnology-enabled immunotherapy can be translated to clinical practice for esophageal cancer. Importantly, while a wealth of innovative nanoplatforms have shown remarkable success in modulating the TME of other solid tumors, a significant translational gap remains. Future research must prioritize the rigorous evaluation of these nanomedicines specifically within orthotopic and genetically engineered models of esophageal cancer to account for its unique physiological and immunological barriers. The heterogeneity of the TME between patients and within individual tumors necessitates personalized approaches that can adapt to specific TME characteristics (14, 21). The development of biomarkers for patient stratification and treatment monitoring is essential for optimizing therapeutic outcomes (21, 98). Scalable manufacturing processes and quality control measures must be established to ensure consistent production of nanomedicines (16, 113).

The potential toxicity of nanomaterials, particularly metal-based nanoparticles, requires careful evaluation through comprehensive preclinical and clinical studies (20, 113). Long-term biocompatibility, biodegradability, and clearance mechanisms must be characterized to ensure patient safety (20, 113). The immunogenicity of nanoparticle components, including synthetic polymers and surface ligands, may trigger adverse immune responses that limit therapeutic efficacy (16, 113).

Artificial intelligence and machine learning approaches offer opportunities for optimizing nanoparticle design and predicting therapeutic outcomes (106, 108, 114). AI-guided design can accelerate the development of nanocarriers with optimal physicochemical properties for specific TME characteristics (106, 114). Integration of spatial proteomics and transcriptomics with nanotechnology can provide insights into TME dynamics and guide personalized treatment strategies (98).

Future research should focus on developing multifunctional nanoplatforms that can simultaneously monitor TME changes and deliver therapeutic interventions in a closed-loop manner (114, 115). Theranostic nanoparticles that combine imaging and therapeutic capabilities can enable real-time assessment of treatment response and adaptive therapy (114, 115). The development of TME-responsive nanomedicines that can adapt their properties in response to dynamic changes in the TME represents an exciting frontier for precision cancer immunotherapy.

In conclusion, nanotechnology-enabled immunomodulation represents a highly promising conceptual approach for overcoming therapeutic barriers in esophageal cancer. By targeting the immunosuppressive TME with precision-engineered nanocarriers, it is possible to enhance antitumor immunity, overcome resistance mechanisms, and improve clinical outcomes. Continued interdisciplinary collaboration between materials scientists, immunologists, and clinicians will be essential for translating these promising approaches from the laboratory to the clinic, ultimately benefiting patients with esophageal cancer.
